# Respiratory viral infections among hospitalized adults: experience of a single tertiary healthcare hospital

**DOI:** 10.1111/irv.12237

**Published:** 2014-02-03

**Authors:** Ellie Walker, Michael G Ison

**Affiliations:** a Division of Infectious Diseases, Northwestern University Feinberg School of MedicineChicago, IL, USA; b Division of Organ Transplantation, Northwestern University Feinberg School of MedicineChicago, IL, USA

**Keywords:** Epidemiology, infection, outcomes, respiratory, virus

## Abstract

**Background:**

Following the 2009 H1N1 pandemic, there have been a large number of studies focusing on the epidemiology and outcomes of influenza A infection; however, there have been fewer studies focused on other respiratory viral infections.

**Objectives:**

To define the epidemiology and outcomes of non-influenza respiratory viral infections in hospitalized adults.

**Patients/Methods:**

Data on all patients ≥18 years of age with a positive molecular respiratory viral assay who were hospitalized at a single tertiary healthcare system in Chicago, IL, from retrospectively collected and analyzed.

**Results:**

Over the study period, 503 of 46 024 (1·1%) admitted patients had a positive RVP result. Human rhinovirus was the most commonly detected virus followed by influenza A, human metapneumovirus, respiratory syncytial virus, and parainfluenza virus, adenovirus, and influenza B, respectively. Infection in immunocompromised patients was associated with a higher rate of progression to pneumonia and death.

**Conclusions:**

Non-influenza respiratory viral infections are commonly detected among adults admitted to the hospital and can cause serious illness. The data can inform the prioritization of research into novel antiviral therapies for these infections.

## Introduction

Respiratory viral infections are a common cause of respiratory tract infection in both healthy and immunocompromised adults resulting in a large number of hospital admissions as well as significant morbidity and mortality. Studies have shown that up to one-third of cases of community-acquired pneumonia (CAP) among hospitalized adults are caused by respiratory viruses.^1^ The recent 2009 influenza A/H1N1 pandemic increased awareness of the importance of respiratory viral infections among hospitalized patients, and there have been a growing number of studies focused on the epidemiology and outcome of influenza infection among hospitalized adults.^1^–^4^ Influenza is a major contributor to morbidity and mortality globally; however, far fewer studies have focused on the impact of the wide range of other respiratory viruses in hospitalized adults.^5^

Prior studies frequently had two common limitations: Few symptomatic hospitalized adults were screened for respiratory viruses beyond influenza and screening often relied on assays which lack the sensitivity of contemporary molecular techniques. The need to accurately discriminate between influenza subtypes due to the emergence of mutations conferring oseltamivir resistance generated enhanced demand for molecular diagnostics for respiratory viruses. This demand coincided with the broader availability of a range of commercially developed, FDA-approved molecular assays that had the ability to identify a wide range of respiratory viruses. Although the sensitivity and the specificity of the molecular assays vary by specimen type and virus of interest, they are generally more sensitive than culture and antigen-based assays for the detection of most respiratory viruses.^6^

During the pandemic of 2009–2010, most hospitalized patients at Northwestern Memorial Hospital with fever and/or respiratory symptoms had respiratory specimens collected and tested using a molecular assay. We therefore attempted to use these data to define the epidemiology and outcome of non-influenza respiratory viral infections on adults hospitalized at our single center.

## Methods

After institutional review board (IRB) approval, a retrospective chart review of all patients with positive xTAG respiratory viral panel (RVP; Luminex, Austin, TX, USA) or ProFlu+ (Gene-Probe, San Diego, CA, USA) testing who were hospitalized at Northwestern Memorial Hospital (NMH) and Prentice Women's Hospital was conducted. NMH and Prentice Women's Hospital are part of a large, urban, tertiary healthcare system in Chicago, Illinois. NMH includes all adult medical and surgical wards as well as all adult intensive care units; the hospital has one of the highest volume solid organ transplant programs in the United States. Prentice Women's Hospital includes all inpatient oncology wards, including one ward for the treatment of hematopoietic stem cell transplant recipients, and obstetrics and gynecology wards.

A list of all patients who had a positive RVP or ProFlu+ assay performed at NMH or Prentice Women's Hospital during the study period was obtained from the hospital's diagnostic and molecular biology laboratory. Only patients 18 years of age and older who were admitted to NMH or Prentice Women's Hospital between April 1, 2009, and March 31, 2010, and had positive testing for respiratory viral infections with either the ProFlu+ or RVP were included in the study. The electronic medical records of included patients were reviewed by one of the authors; the hospital only utilized electronic medical records. Patients who were admitted more than once during the study period were included with each admission's data recorded separately.

Assays were sent on admitted patients at the discretion of the admitting clinician with the exception of patients admitted to the ICU. It was hospital policy to obtain an RVP from every patient on admission or transfer to the ICU; patients remained in isolation until testing returned negative or until approved to discontinue isolation by infection control for patients with positive assays. The ProFlu+ assay identifies influenza A, influenza B, and RSV and is applied to nasal swabs. ProFlu+ was generally only run on patients when testing was ordered from a clinic or from patients without underlying medical conditions evaluated in the emergency department. From prior studies, the ProFlu+ assay is highly sensitive and specific (sensitivity = 100%, 97·8%, and 89·5%; specificity = 92·6%, 98·6%, and 94·9% for influenza A, influenza B, and RSV, respectively).^7^ The RVP identifies and subtypes influenza A/H1N1, influenza A/H3N2, influenza B, respiratory syncytial virus (RSV) A and B, parainfluenza virus (PIV) 1–3, human metapneumovirus (hMPV), rhinovirus (hRV), and adenovirus (AdV). The RVP can be applied to nasal swabs, nasal washes, and specimens from the lower airway, including bronchoalveolar lavage (BAL) and non-bronchoscopic bronchoalveolar lavage (NBBAL); see Table 1 for sensitivity and specificity data for both the ProFlu+ and RVP assays.^8^

**Table 1:** Clinical performance of ProFlu+ and RVP assays^7^,^8^

Virus	RVP		ProFlu+	
	
	Sensitivity (%)	Specificity (%)	Sensitivity (%)	Specificity (%)
Influenza A	97·1	96·6	100	92·6
H1 subtype of influenza A	100	99·8	–	–
Influenza B	90·3	97·8	97·8	98·6
RSV	–	–	89·5	94·9
RSV A	95·9	98·4		
RSV B	97·6	98·3		
PIV 1	97·8	99·7		
PIV 2	90·4	99·5		
PIV 3	91·9	99·8		
hRV/enterovirus	95·5	83·6		
AdV	82·5	99·3		
hMPV	95·3	95·9		

AdV, adenovirus; hMPV, human metapneumovirus; PIV, parainfluenza virus; hRV, human rhinovirus; RSV, respiratory syncytial virus.

During the study period, viral culture and rapid antigen testing were not utilized in patients admitted to the hospital. Rapid antigen testing was utilized in patients seen in the emergency department, but additional molecular testing was sent if patients were subsequently admitted or if results were negative. In addition, during the study period, all patients admitted to the medical intensive care unit for any indication had nasal swabs and/or BAL specimens sent for the RVP. Patients who were less than 18 years of age at the time of hospitalization and patients who had molecular testing but were not hospitalized were excluded from the study.

Extracted data included presenting symptoms, comorbid medical conditions, location of care, treatment received, and outcome. Patients were considered to be immunocompromised if they were affected by any of the following conditions: hematologic malignancy, solid tumor malignancy, autoimmune or rheumatologic disease, asplenia, primary immunodeficiency, or were the recipient of a solid organ transplant. Patients were also considered to be immunocompromised if they had received a hematopoietic stem cell transplant (HSCT) within the year prior to their hospital admission, if they had received chemotherapy in the 30 days prior to admission, or if they were taking chronic immunosuppressive medications. Data on the presence of both respiratory and non-respiratory co-infection were also collected. Co-infection was defined as infection due to bacterial and/or fungal pathogens. Co-infection was considered to be present if there was microbiologic evidence of infection, radiographic evidence of infection (e.g., an abscess seen on imaging), or clinical suspicion based on physician documentation in the electronic medical record. Microbiologic evidence of infection included culture data, positive antigen testing (e.g., *Streptococcus pneumoniae* antigen), and polymerase chain reaction (PCR) testing (e.g., *Clostridium difficile* PCR). Data on co-infecting pathogens were collected for cases in which microbiologic evidence of co-infection was present.

Severity of illness was assessed by examining several factors including need for intensive care unit (ICU)-level care, need for mechanical ventilation, need for vasopressor support as well as patient outcome. Descriptive statistics were calculated, and the Pearson's chi-squared or Fisher exact test was used to compare proportions.

## Results

During the study period, 46 024 patients were admitted to Northwestern Memorial Hospital and Prentice Women's Hospital and approximately 3500 patients had molecular respiratory viral diagnostics performed. Of these, 502 (1·1% of hospitalized patients; 14·4% of those with molecular testing) had a positive result and were included in the study. A total of 26 patients were tested with the ProFlu+ assay. Twenty-four patients tested positive for influenza A and two patients tested positive for RSV by ProFlu+. The remainder of patients were tested using the RVP.

**Figure 1:**
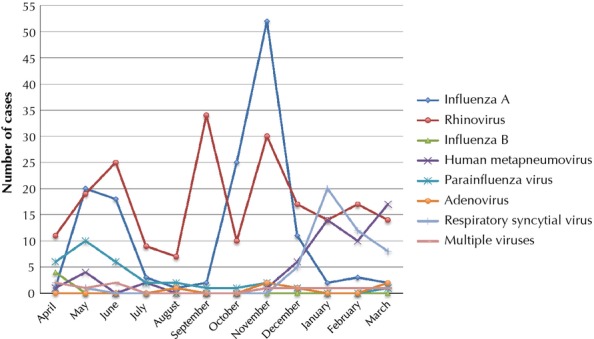
Seasonal distribution of viruses detected in this study.

**Figure 2:**
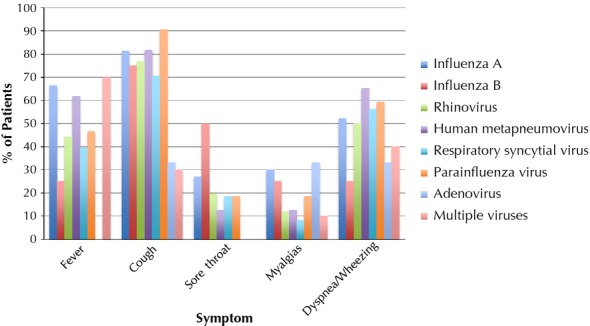
Presenting symptoms by virus type.

The most frequently detected viruses were hRV (207), 2009 influenza A/H1N1 (140), hMPV (55) followed by RSV (48), PIV (32), adenovirus (6), and influenza B (4); 10 patients had multiple viruses detected. Patient characteristics can be found in Table 2. Nearly half of the patients were immunocompromised, and over a third had pre-existing lung disease. Data of the seasonality of each virus and presenting symptoms are presented in Figures1 and 2, respectively. Nearly 80% of patients received antibacterial therapies during their illness, and approximately a quarter of all patients required ICU care during their hospitalization (Table 3).

**Table 2:** Demographic features of hospitalized patients with detectable respiratory viruses

	Total	AdV	Influenza B	Influenza A/H1N1	hMPV	Multiple viruses*	PIV	hRV	RSV
Number of cases	502	6 (1%)	4 (1%)	140 (28%)	55 (11%)	10 (2%)	32 (6%)	207 (41%)	48 (10%)
Age									
18–19	7 (1%)	0	0	2 (1%)	1 (2%)	0	0	4 (2%)	0
20–29	71 (14%)	1 (17%)	2 (50%)	38 (27%)	2 (4%)	1 (10%)	5 (16%)	19 (9%)	3 (6%)
30–39	83 (17%)	2 (33%)	0	32 (23%)	2 (4%)	2 (20%)	2 (6%)	36 (17%)	7 (15%)
40–49	77 (15%)	2 (33%)	0	17 (12%)	12 (22%)	1 (10%)	3 (9%)	42 (20%)	0
50–59	96 (19%)	1 (17%)	1 (25%)	32 (23%)	11 (20%)	3 (30%)	6 (19%)	35 (17%)	7 (15%)
60–69	90 (18%)	0	1 (25%)	15 (11%)	15 (27%)	2 (20%)	10 (31%)	36 (17%)	11 (23%)
70–79	42 (8%)	0	0	4 (3%)	7 (13%)	1 (10%)	4 (13%)	20 (10%)	6 (13%)
80–89	31 (6%)	0	0	0	5 (9%)	0	2 (6%)	12 (6%)	12 (25%)
90+	5 (1%)	0	0	0	0	0	0	3 (1%)	2 (4%)
BMI**									
<20	49 (10%)	1 (17%)	0	9 (6%)	1 (2%)	1 (10%)	3 (9%)	27 (13%)	7 (15%)
20–24·99	118 (24%)	1 (17%)	1 (25%)	32 (23%)	14 (25%)	3 (30%)	10 (31%)	47 (23%)	10 (21%)
25–29·99	112 (22%)	2 (33%)	1 (25%)	32 (23%)	13 (24%)	1 (10%)	5 (16%)	46 (22%)	12 (25%)
30–34·99	77 (15%)	1 (17%)	1 (25%)	12 (9%)	12 (22%)	2 (20%)	8 (25%)	33 (16%)	8 (17%)
35–39·99	43 (9%)	0	1 (25%)	12 (9%)	5 (9%)	1 (10%)	3 (9%)	20 (10%)	1 (2%)
≥40	55 (11%)	0	0	9 (6%)	8 (15%)	2 (20%)	2 (6%)	25 (12%)	9 (19%)
Gender = female	291 (58%)	3 (50%)	1 (25%)	82 (59%)	30 (55%)	5 (50%)	21 (66%)	118 (57%)	31 (65%)
Underlying medical conditions									
Documented lung disease	167 (33%)	1 (17%)	1 (25%)	40 (29%)	14 (25%)	3 (30%)	12 (38%)	78 (38%)	18 (38%)
Liver disease	13 (3%)	0	0	2 (1%)	1 (2%)	0	0	8 (4%)	2 (4%)
Renal disease	96 (19%)	1 (17%)	1 (25%)	13 (9%)	16 (29%)	3 (30%)	8 (25%)	39 (19%)	15 (31%)
Diabetes	114 (23%)	0	1 (25%)	22 (16%)	21 (38%)	3 (30%)	8 (25%)	44 (21%)	15 (31%)
Cardiovascular disease	107 (21%)	0	1 (25%)	21 (15%)	17 (31%)	3 (30%)	11 (34%)	39 (19%)	15 (31%)
Pregnant	39 (8%)	1 (17%)	0	30 (21%)	0	0	0	7 (3%)	1 (2%)
Immunocompromised (any cause)	222 (44%)	4 (67%)	3 (75%)	42 (30%)	26 (47%)	5 (50%)	19 (59%)	104 (50%)	19 (40%)
Hematologic malignancy	83 (17%)	1 (17%)	0	16 (11%)	10 (18%)	1 (10%)	6 (19%)	38 (18%)	11 (23%)
Solid tumor	28 (6%)	1 (17%)	1 (25%)	2 (1%)	4 (7%)	1 (10%)	2 (6%)	17 (8%)	0
Autoimmune/rheumatologic condition	27 (5%)	1 (17%)	1 (25%)	6 (4%)	6 (11%)	0	4 (13%)	4 (2%)	5 (10%)
Solid organ transplant	39 (8%)	1 (17%)	0	8 (6%)	4 (7%)	3 (30%)	4 (13%)	17 (8%)	2 (4%)
Chemotherapy within last 30 days	60 (12%)	2 (33%)	0	8 (6%)	9 (16%)	1 (10%)	3 (9%)	28 (14%)	9 (19%)
Stem cell transplant within last 1 year	29 (6%)	1 (17%)	0	7 (5%)	4 (7%)	1 (10%)	1 (3%)	11 (5%)	4 (8%)
HIV	29 (6%)	2 (33%)	1 (25%)	3 (2%)	2 (4%)	0	2 (6%)	18 (9%)	1 (2%)
Asplenic	8 (2%)	0	0	2 (1%)	0	0	0	5 (2%)	1 (2%)
Common variable immunodeficiency	1 (0·2%)	0	0	0	0	0	0	1 (0·5%)	0
Hypogammaglobulinemia	1 (0·2%)	0	0	1 (1%)	0	0	0	0	0

AdV, adenovirus; hMPV, human metapneumovirus; PIV, parainfluenza virus; hRV, human rhinovirus; RSV, respiratory syncytial virus

* Two patients with FluA/H1N1 + hRV, one patient with FluA/H1N1 + hMPV, one patient with FluA/H1N1 + PIV, two patients with hRV + hMPV, two patients with hRV + RSV, one patient with hRV + adenovirus, one patient with hRV + PIV.

** Body mass index (BMI) was excluded for pregnant patients and was not available for all patients.

**Table 3:** Treatment and outcomes of hospitalized patients with detectable respiratory viruses

	Total	AdV	Influenza B	Influenza A/H1N1	hMPV	Multiple viruses	PIV	hRV	RSV
Total cases	502	6 (1%)	4 (1%)	140 (28%)	55 (11%)	10 (2%)	32 (6%)	207 (41%)	48 (10%)
Reason for admission									
Respiratory	337 (67%)	3 (50%)	2 (50%)	104 (74%)	43 (78%)	7 (70%)	23 (72%)	124 (60%)	31 (65%)
Non-respiratory	165 (33%)	3 (50%)	2 (50%)	36 (26%)	12 (22%)	3 (30%)	9 (28%)	83 (40%)	17 (35%)
Abnormal lung imaging	173	3 (50%)	0	40 (29%)	29 (53%)	5 (50%)	15 (47%)	60 (29%)	21 (44%)
Antibiotic therapy	399 (79%)	6 (100%)	4 (100%)	88 (63%)	52 (95%)	10 (100%)	28 (88%)	166 (80%)	45 (94%)
Documented/suspected respiratory co-infection (total)	197 (39%)	1 (17%)	0	41 (29%)	31 (56%)	4 (40%)	12 (38%)	82 (40%)	26 (54%)
Bacterial	181 (36%)	1 (17%)	0	40 (29%)	29 (53%)	4 (40%)	11 (34%)	71 (34%)	25 (52%)
Fungal	16 (3%)	0	0	1 (1%)	2 (4%)	0	1 (3%)	11 (5%)	1 (2%)
Non-respiratory co-infection	84 (17%)	3 (50%)	2 (50%)	16 (11%)	8 (14%)	2 (20%)	7 (22%)	38 (18%)	8 (17%)
Antiviral therapy	183 (36%)	2 (33%)	2 (50%)	119 (85%)	11 (20%)	5 (50%)	6 (19%)	33 (16%)	5 (10%)
ICU admission	125 (25%)	2 (33%)	1 (25%)	29 (21%)	16 (29%)	5 (50%)	7 (22%)	50 (24%)	15 (31%)
Mechanical ventilation	66 (13%)	1 (17%)	0	11 (8%)	12 (22%)	4 (40%)	7 (22%)	23 (11%)	8 (17%)
Non-invasive ventilation	43 (9%)	0	0	7 (5%)	8 (14%)	1 (10%)	5 (16%)	13 (6%)	9 (19%)
Vasopressor	48 (10%)	2 (33%)	0	11 (8%)	6 (11%)	2 (20%)	3 (9%)	20 (10%)	4 (8%)
New renal replacement therapy	18 (4%)	0	0	2 (1%)	2 (4%)	2 (20%)	1 (3%)	7 (3%)	4 (8%)
Discharge diagnosis									
Pneumonia	157 (31%)	3 (50%)	0	34 (24%)	28 (51%)	3 (30%)	11 (34%)	64 (31%)	14 (29%)
Lung disease exacerbation	98 (20%)	0	1 (25%)	16 (11%)	8 (14%)	2 (20%)	6 (19%)	52 (25%)	13 (27%)
Outcome/discharge									
home	434 (86%)	4 (67%)	4 (100%)	130 (93%)	46 (84%)	8 (80%)	24 (75%)	180 (87%)	38 (79%)
Other medical facility	44 (9%)	0	0	2 (1%)	8 (14%)	1 (10%)	6 (19%)	20 (10%)	7 (15%)
Death/hospice	24 (5%)	2 (33%)	0	8 (6%)	1 (2%)	1 (10%)	2 (6%)	7 (3%)	3 (6%)

AdV, adenovirus; hMPV, human metapneumovirus; PIV, parainfluenza virus; hRV, human rhinovirus; RSV, respiratory syncytial virus.

Patients with non-influenza infections and influenza infection had approximately the same need for ICU admission (26% versus 21%, *P* = 0·23) and vasopressor use (10% versus 8%, *P* = 0·60). The need for mechanical ventilation was higher among patients with non-influenza infection than among patients with influenza infection (15% versus 8%, *P* = 0·03). The rate of death or discharge with hospice was low overall and approximately the same in patients with non-influenza versus influenza infection (4% versus 6%, *P* = 0·38).

Patients with hematologic malignancy had a higher rate of ICU admission (43·4% versus 21%, *P* < 0·001), need for ventilator support (25% versus 10·7%, *P* < 0·001), and need for vasopressor support (30% versus 5·5%, *P* < 0·001) as well a higher rate of death or discharge with hospice care (20·5% versus 1·7%, *P* < 0·001) when compared to other groups. Of the patients with hematologic malignancy who had undergone HSCT within the year prior to admission, the date of death or discharge with hospice was greater, but not statistically significant (34·8% versus 20·5%, *P* = 0·104).

Approximately 39% of patients included in the study were obese (BMI ≥30). Of all patients with non-influenza infection, approximately 39% were obese. The data did not suggest that obesity was a risk factor for severe non-influenza infection. The rates of the following markers of disease severity did not differ significantly between obese and non-obese patients, respectively: (i) ICU admission (46·7% versus 53·2%, *P* = 0·099), (ii) need for mechanical ventilation (45·3% versus 54·7%, *P* = 0·386), (iii) need for vasopressor support (37% versus 63%, *P* = 1), (iv) outcome of death/discharge with hospice care (33% versus 67%, *P* = 0·84).

Patients with underlying lung disease had a higher rate of need for ICU admission (32% versus 21% *P* = 0·007), but did not have a significantly higher need for mechanical ventilation than patients without underlying lung disease.

### Rhinovirus

Twenty percent (10/50) of patients requiring ICU admission and nearly 40% (9/23) of those requiring mechanical ventilation had positive samples for rhinovirus from the lower airway. Of the patients who required ICU admission, over half had a documented or suspected respiratory co-infection. One-half of patients (26/50) who required ICU care had underlying immune compromise and nearly two-thirds of these patients (15/26) had a hematologic malignancy.

Although most rhinovirus-infected patients were discharged home, seven died or were discharged to hospice and 20 were discharged to a long-term care rehabilitation facility. Of the fatal cases, five had documented or suspected co-infection, five had hematologic malignancy, and three patients had both hematologic malignancy and co-infection. Two hematopoietic stem cell transplant (HSCT) recipients with rhinovirus infection died.

### Influenza A virus

Twenty-nine patients (20·7%) required ICU-level care. Approximately one-third (9/29) of patients who required ICU-level care and over one-third (8/11) of those who required mechanical ventilation had BAL or NBBAL samples which were positive for influenza A/H1N1. Of those who required ICU-level care, 16 patients had a known or suspected non-viral respiratory co-infection. An additional six patients who required ICU-level care had a non-respiratory co-infection including bacteremia, *Clostridium difficile* infection, and abscess. Nearly one-third (9) of patients infected with influenza A who required ICU care had a hematologic malignancy.

A total of 119 (85%) patients received oseltamivir during admission. Three of these patients received oseltamivir initially and were then transitioned to another neuraminidase inhibitor (two peramivir and one zanamivir). Four patients received multiple antiviral medications in a regimen that included oseltamivir.

Exacerbation of underlying lung disease and bacterial pneumonia were the most common discharge diagnoses. The majority of patients were discharged home. Eight patients died during hospitalization or were discharged with hospice. Of these fatal cases, 6 (75%) had a documented or suspected co-infection, 7 (88%) had underlying immunocompromise, and 6 (75%) had hematologic malignancy.

### Influenza B virus

Three of the four patients with influenza B infection had underlying immunocompromise (SLE on chronic steroids, HIV, solid tumor). One patient required ICU-level care. None of these patients required mechanical ventilation, vasopressor support, or non-invasive positive pressure ventilation. No patients were suspected to have a respiratory co-infection, and none of the four patients had abnormal lung imaging; however, all four patients received antimicrobial therapy during admission (one patient received empiric antibiotics for sepsis). Two patients received oseltamivir. All four patients were discharged home.

### Human metapneumovirus

Nearly 53% of patients with hMPV infection had lung imaging that was consistent with pneumonia, yet 30% (8/29) of these patients had neither suspected nor documented respiratory co-infection with a bacterial or fungal copathogen. Almost one-third of patients (16/55) required ICU care, six patients required vasopressor support, 12 required mechanical ventilation, and eight patients required NIPPV; only 5 of these 16 patients had NBBAL or BAL samples positive for hMPV. The majority of patients were discharged home (46/55); eight patients required transfer to another medical facility. Only one hMPV-infected patient died. This patient had a pre-existing hematologic malignancy; their course was complicated by bacterial pneumonia, bacteremia, and fungemia.

### Respiratory syncytial virus

Of the 15 patients who required ICU-level care, eight patients required mechanical ventilation, nine required NIPPV, and four required vasopressor support. Five patients who required both ICU-level care and ventilator support had BAL or NBBAL samples which were positive for RSV. One patient who required ICU admission also had a methicillin-resistant *Staphylococcus aureus* (MRSA) bacteremia. Three patients received IV immunoglobulin (IVIG). The most common discharge diagnoses were exacerbation of underlying lung disease and bacterial pneumonia. Two patients died and one patient was discharged with hospice care. All three of these patients had documented or suspected bacterial pneumonia. Additionally, one of these three patients had a documented bacteremia and *C. difficile* infection.

### Parainfluenza virus

A total of seven patients required ICU-level care; seven patients required mechanical ventilation; three patients required vasopressor support; and five patients required NIPPV. Two patients who required both ICU-level care and mechanical ventilation had BAL or NBBAL samples positive for PIV. Over 80% of patients were discharged home; six patients were transferred to another medical facility and two patients died. One of the two patients who died had suspected fungal pneumonia; both patients had hematologic malignancy.

### Adenovirus

Cough and dyspnea were among the reported symptoms on admission; however, only half of patients had respiratory complaints on presentation. Four patients had underlying immunocompromise and two required ICU-level care (HIV and MDS). One of these two patients had documented bacterial pneumonia as well as bacteremia and the other had a bacteremia; both of these patients died.

### Multiple respiratory viruses

A total of 10 patients were diagnosed with multiple respiratory viruses during admission. A description of each patient's RVP results is provided in Table 2. Our data suggest that infection with multiple viruses may lead to more severe infection; however, the small number of patients in this group did not allow for meaningful statistical analysis to be performed.

Half of these patients had underlying immunocompromise. Five patients required ICU-level care, and four required mechanical ventilation. All patients received antimicrobial therapy during admission. All patients with influenza A in addition to another respiratory virus received antiviral therapy with either oseltamivir or zanamivir during admission. The most common discharge diagnosis was exacerbation of underlying lung disease. Nine of ten patients were discharged home or to another facility. One patient, who had H1N1 and PIV infection, a documented bacterial pneumonia, and hematologic malignancy, died.

## Discussion

To date, many studies have described the epidemiology and outcome of 2009 pandemic influenza A/H1N1 in hospitalized adults, but few studies focused on other respiratory viruses in this unique population.^2^,^9^–^11^ Our study is one of the largest single-center studies to describe the epidemiology and outcomes of non-influenza respiratory viruses in hospitalized patients. Our data provide evidence that non-influenza infection contributes significantly to inpatient admissions for respiratory illness and may cause or contribute to severe disease, particularly in patients with underlying lung disease or compromised immunity.

Overall, the majority of patients in our study were infected with non-influenza respiratory viruses (Table 1). As expected, influenza and RSV infections peaked in the winter months and hRV predominated in the fall.^12^,^13^ Consistent with past studies, hRV and influenza A were the most commonly detected viruses, while the overall incidence of other respiratory viruses was relatively low.^1^,^14^ Influenza infection is well recognized as the cause of significant healthcare costs, morbidity, and mortality in the United States; however, the role of non-influenza respiratory viruses is not as well appreciated.^15^ The high proportion of non-influenza infection in this study (72%) underscores the importance of non-influenza viral infection in contributing to severe illness necessitating hospital and ICU admission in adults, as has been found by others.^16^ Rates of mortality were also similar to other studies; unfortunately, the number of fatal cases in our series was too small to allow for meaningful statistical comparison.^16^

Our study found a similar rate of need for ICU admission, vasopressor use, and outcome of death or discharge with hospice care between patients with influenza infection and those with non-influenza infection. The need for mechanical ventilation was higher among patients with non-influenza infection than among patients with influenza infection. Nonetheless, most patients who required ventilator support had a suspected or documented bacterial or fungal respiratory co-infection. This suggests that co- or superinfection may be a predictor of more severe disease, when identified, as has been found in other studies.^17^,^18^ Likewise, infection with some viruses (RSV, adenovirus, or multiple viruses) may also be associated with a higher rate of critical illness.

Several studies have investigated the incidence and clinical consequences of mixed respiratory viral infection (MRVI). A recent meta-analysis of eight studies examining the incidence of MRVI revealed approximately a 5% incidence of dual respiratory virus infection (range: 1·8% to 15·8%).^19^ Although we only documented MRVI in 2%, cases were more common among individuals with underlying lung disease. Progression to lower airway involvement appears to be more common among patients with MRVI (30% in this study), confirming the findings of the recent meta-analysis, where 40% developed LRT infection.^19^ Although most other studies demonstrated higher rates of mortality among those with MRVI, only 1 in 10 MRVI patients in this study died; this patient had underlying immunocompromise and bacterial pneumonia in addition to H1N1 influenza A and PIV MRVI.^20^,^21^ This study had few patients with MRVI, limited the ability to perform detailed statistical analyses.

There is evidence that severe RSV infection with lower respiratory tract involvement can occur, in particular in the elderly or those with underlying medical conditions.^22^ Overall, the severity of illness in RSV approaches that of influenza A/H1N1 in our study with similar or higher rates of need for ICU admission (31% versus 21%), ventilator support (17% versus 8%), and death/discharge with hospice care (6% versus 6%). A recently published study found a greater number of annual cases of RSV than in our study. Similar to our findings, this study was able to demonstrate manifestations, complications, and outcomes of RSV infection comparable to those of seasonal influenza among hospitalized adults.^23^

As with the other viruses studied, there was a large proportion of patients with hMPV with underlying immunocompromise and/or lung disease. Human metapneumovirus has been shown to cause more severe illness in immunocompromised patients.^24^,^25^ The single patient with hMPV infection who died in our study had underlying hematologic malignancy in addition to multiple co-infections. Pneumonia was the most common discharge diagnosis and lung disease exacerbation was the second most common discharge diagnosis among patients with hMPV infection. Several case reports support our finding of severe hMPV infection among immunocompromised patients.^24^,^25^

Although a large proportion of patients had hRV infection detected (41%), it is unclear what role this virus played in their hospital course. Human rhinovirus can be shed for a prolonged period of time. Further, the study did not include a cohort of patients admitted without respiratory virus detected; as a result, the exact role hRV is playing in the individual patient's hospitalization cannot be clearly understood. Nonetheless, hRV has been implicated in causing pneumonia in immunocompromised patients, elderly patients and is known to contribute significantly to exacerbation of lung disease in patients with asthma and COPD.^26^–^32^ It is therefore possible that the identified hRV is playing a role in some or all of the patients' hospitalizations. Future case–control or prospective studies will be needed to fully define the role of hRV in hospitalized patients.

A large proportion of patients in our study had compromised immunity (Table 2). It is possible that the high proportion of patients with underlying immunocompromise reflects the large population of patients with malignancies, solid organ transplant recipients, and HSCT recipients served by our hospital. Nonetheless, less than 10% of our overall hospital admissions relate to this population, whereas 44% of admitted patients included in our study had underlying immune compromise, suggesting that this population is disproportionately severely affected by respiratory viruses. This finding is consistent with other studies, which have suggested that respiratory viruses cause a disproportionately high rate of hospitalization in immunocompromised adults.^30^,^32^–^35^

Previous studies have demonstrated that more severe respiratory viral illness may occur in patients with compromised immunity.^30^,^32^,^36^ Influenza is the most well-studied respiratory viral infection among immunocompromised patients. More severe infections, including pneumonia and an increased risk of bacterial or fungal superinfection, have been described in immunocompromised patients with influenza.^31^,^32^,^37^–^39^ The effect of non-influenza respiratory viral infection has been less well described in this patient population. Our study suggests that non-influenza viruses contribute significantly to the need for hospitalization and ICU admission among immunocompromised patients. Infection with non-influenza respiratory viruses caused a wide range of respiratory illness from mild upper respiratory tract infection to severe illness, necessitating ICU admission and mechanical ventilation.

Intensive care was needed in approximately 25% of patients overall and was more commonly needed among immunocompromised adults. Overall, patients with hematologic malignancy had a higher rate of need for ICU admission, mechanical ventilation, vasopressor support, and a higher rate of death or discharge with hospice care when compared to other groups. This is likely related to the increased risk of progression to lower respiratory tract infection with high mortality in patients undergoing treatment for hematologic malignancies, especially recipients of HSCT.^40^–^42^ As was the case in past studies, the cause of pneumonia in these patients could often not be determined due to the lack of microbiologic data from lower respiratory tract specimens. Immunocompromised individuals were also at increased risk of co-infection with a bacterial, fungal, or viral pathogen as has been demonstrated in prior studies, making it difficult to determine to what degree a poor outcome may have been independently related to the underlying respiratory viral infection.

Given that respiratory viruses can be spread efficiently among hospitalized immunocompromised patients populations, clinicians should have a low index of suspicion of respiratory viral infections in these patients, even when symptoms develop during the course of hospitalization. Infection control measures, including use of droplet precautions, should be instituted as soon as a respiratory viral infection is suspected in these vulnerable patients.

Patient characteristics known to be associated with severe influenza (e.g., underlying immunocompromise and chronic lung disease) were assessed for as was the presence of other medical comorbidities including obesity. Recent studies have suggested that obesity may be an independent risk factor for influenza A, and several studies have found an association between obesity and ICU admission and/or death in H1N1 infection.^2^,^43^–^46^ Of the 454 patients with BMI data in our study, 175 (∼38·5%) were obese (BMI ≥30). Our study does not suggest that obesity is a risk factor for more severe illness in non-influenza infection. There were no statistically significant differences when comparing the rates of need for ICU admission, mechanical ventilation, or vasopressor use between obese and non-obese patients with non-influenza infection. In addition, there was no significant difference in the rate of death or discharge with hospice care between obese and non-obese patients with non-influenza infection. Given the small numbers, we were unable to perform a multivariable analysis to assess the role of obesity with regard to these factors.

Approximately 33% of patients had underlying lung disease. Overall, exacerbation of underlying lung disease was the second most common discharge diagnosis, and non-influenza respiratory viruses, especially RSV, rhinovirus, and PIV, were responsible for a significant proportion of these exacerbations. This is consistent with previous studies examining the epidemiology of respiratory viruses in exacerbation of underlying lung disease.^47^ The role of influenza A in causing exacerbation of lung disease and severe respiratory illness is well recognized; however, the role of non-influenza respiratory viruses in respiratory illness has been less well studied.^4^,^26^,^27^,^43^,^48^–^50^ Non-influenza respiratory viruses were associated with a higher rate of need for ventilator support in patients with a diagnosis of exacerbation of airways disease than influenza A infection.

The overwhelming majority of patients (400/502) received antibacterial therapy, but less 40% of patients who received antibacterial therapy were discharged with a diagnosis of bacterial pneumonia. This finding may reflect pressure to deliver empiric pneumonia coverage within a prescribed time frame or empiric antimicrobials given as part of early goal-directed therapy; however, it may suggest the need for more judicious use of broad-spectrum antimicrobials. The majority of patients (119/140) with influenza A/H1N1 infection received antiviral therapy with oseltamivir during admission. This rate is consistent with previous studies investigating the rate of antiviral treatment for influenza A during the 2009 pandemic and suggests increased but still suboptimal compliance with recommendations to treat all hospitalized patients with influenza A regardless of the time from symptom onset.^51^,^52^ This is particularly important as previous studies have shown that >40% of patients who are hospitalized with seasonal influenza present >48 hours after symptom onset and that patients who develop critical illness have the longest delay between symptom onset and presentation.^11^,^53^,^54^ Appropriate and early antiviral therapy for influenza has been shown to improve survival in critically ill and hospitalized patients.^55^ In addition, there is evidence that antiviral therapy, even if started greater than 48 hours after symptom onset, improves survival compared to no treatment.^53^,^55^–^57^ Most experts recommend initiation of antiviral therapy for any patient with suspected influenza-like illness on admission to the hospital, even if diagnostic studies are pending.^52^,^58^ While late treatment is superior to no treatment, the best outcomes are seen with the initiation of antiviral therapy within 48 hours of symptom onset.^59^,^60^ If influenza is ruled out or a non-influenza virus is detected, the antiviral can generally be discontinued.

These findings suggest that more sensitive rapid testing modalities for respiratory viral illnesses are needed. Several rapid influenza tests are currently available but have excellent specificity, but relatively poor sensitivities ranging from 50–70%.^61^ Limited studies demonstrate that rapid testing for influenza decreased unnecessary empiric antibiotic use, increased the appropriate treatment for influenza A infection, decreased unnecessary use of imaging studies, and decreased length of time to discharge.^62^ The development of rapid testing could detect multiple respiratory viruses and may therefore improve patient outcomes and decrease healthcare costs. Novel molecular techniques promise to improve sensitivity and provide multivirus testing with improved turnaround times and reduced complexity; however, the availability of molecular tests, particularly in the community, remains limited. Furthermore, even with short turnaround times, time to diagnosis may be prolonged if the diagnostic test is not performed frequently or requires that samples be sent to a reference laboratory.

This study also highlights the importance of developing antiviral agents for the treatment of respiratory viral infections, especially for immunocompromised patients. Given the significant risk of progressive infection and mortality in patients with compromised immune systems, studies of novel agents should include this patient population early in the development process. Unfortunately, these patients are typically excluded in early studies, and as a result, the optimal timing, dose, and duration of the antiviral therapy are often not defined.

This study was limited by the fact that data were collected retrospectively. RVP and ProFlu+ results were given accession numbers that were then linked to patient name and medical record number. Accession numbers were re-used over time and approximately 30 accession numbers could not be matched with the correct patient at the time of data collection. In addition, it was difficult to identify the source of each RVP (upper versus lower respiratory tract) based on the information in the electronic medical record. It is not possible to assess how many cases could have been missed in this study as sensitivity of detection is not only a function of the test characteristics of the assay but also how the samples are collected (i.e., inadequate specimens, which are not clinically assessed for with available molecular diagnostic assays) and which site is sampled (i.e., upper versus lower airway). It was also difficult to assess whether or not bacterial co-infection was present in many cases as appropriate diagnostic specimens were not collected. The RVP assay used during the study period was unable to distinguish hRV from enterovirus; however, the proportion of hRV to other viruses found was not unexpected. Lastly, although utilized only for a limited number of patients admitted to the hospital, patients who had the ProFlu+ were tested for influenza and RSV; as a result, numbers of other respiratory viruses may be slightly underestimated.

Although there has been a significant focus on the epidemiology and outcomes of influenza infection among hospitalized adults, our study provides further evidence that non-influenza respiratory viruses contribute significantly to hospitalization and morbidity in adults. Immunocompromised patients are at increased risk of mortality if infected with these viruses. These data are important in helping to prioritize drug development to address the more common respiratory viral infections in this at-risk population. This study suggests the need for the development of more sensitive rapid tests to detect respiratory viruses, which may in turn decrease unnecessary antibiotic utilization. Future prospective studies are needed to better define the epidemiology and outcomes of these respiratory viral pathogens.
